# Prediction of suicide attempts in depressive disorder and panic disorder with/without agoraphobia: multivariate analysis of clinical predictors

**DOI:** 10.3389/fpsyt.2026.1710182

**Published:** 2026-02-17

**Authors:** Borjanka Batinic, Barbara Kecman, Goran Opacic, Matej Stuhec

**Affiliations:** 1 Faculty of Philosophy, Department of Psychology, University of Belgrade, Belgrade, Serbia; 2 Clinic of Psychiatry, University Clinical Centre of Serbia, Belgrade, Serbia; 3 Department for Pharmacology and Clinical Pharmacy, University of Maribor, Medical Faculty, Maribor, Slovenia

**Keywords:** depressive disorder, multivariate analysis, panic disorder/agoraphobia, prediction, suicide attempts

## Abstract

**Introduction:**

Suicide risk in patients with depressive disorder (DD) and panic disorder with or without agoraphobia (PD/A) is often associated with depression, hopelessness, and anxiety-related factors, but their predictive roles remain unclear.

**Aim:**

To identify the strongest clinical predictors of suicide attempts in patients with DD and PD/A, and to assess the applicability of a predictive model within and across diagnostic groups.

**Methods:**

A total of 94 patients (48 with DD- acute or recurrent, 46 with PD/A) were assessed using the Beck Scale for Suicide Ideation (BSSI), Beck Depression Inventory-II (BDI-II), Beck Hopelessness Scale (BHS), Anxiety Sensitivity Index-Revised (ASI-R), and Beck Anxiety Inventory (BAI). Univariate and multivariate analyses were conducted to evaluate the relative predictive value of these factors for suicide attempts within and across diagnostic groups.

**Results:**

Suicide attempts were reported by 23 patients with DD (48%) and only one patient with PD/A (2.17%). In DD sample, suicidal ideation was the strongest predictor of suicide attempts, followed by depression severity. ROC analyses indicated optimal cut-offs of 11 for BSSI and 35 for BDI-II, yielding high sensitivity (82.6%) and specificity (92%). Hopelessness and anxiety sensitivity did not retain predictive value in multivariate analysis, whereas panic/agoraphobic symptoms showed a suppressor effect, reducing risk by 11%. When applied to the PD/A group, the model estimated an average predicted risk of 9.3%, but failed to identify the single attempter, resulting in zero sensitivity.

**Conclusions:**

High levels of suicidal ideation and depressive symptoms were the strongest predictors of suicide attempts in DD. Panic and agoraphobic symptoms exerted a suppressor effect on suicide risk. In the PD/A group, however, the model’s predictive utility was extremely limited due to the very low number of suicide attempts, and these findings should therefore be considered preliminary.

## 1 Introduction

Research on panic attacks and panic disorder (with or without agoraphobia) as predictors of suicidality has yielded contradictory findings. While some longitudinal studies, such as that by Warshaw et al. (1), found no direct association between panic disorder and suicidal behavior in the absence of additional risk factors, broader analyses offer a more nuanced picture. For instance, Bentley et al. (2) reported in a meta-analysis that anxiety disorders, including panic disorder, tend to be relatively weak long-term predictors of suicidal ideation and behavior. Clinically, individuals with panic disorder often exhibit heightened concern about their health and an intense fear of death, along with frequent help-seeking behavior, which may make them less prone to suicidal behavior. Some studies suggest that the link between panic disorder and suicidality is primarily mediated by comorbid depressive disorders (3, 4), and that panic disorder alone does not elevate the risk of suicide attempts (5). Conversely, other research indicates that panic disorder is independently associated with an increased risk of suicidal ideation and attempts, even in the absence of depression or other comorbid conditions (6–8). A recent systematic meta-analysis suggests that the prevalence of suicidal ideation and suicide attempts is notably elevated in individuals with panic disorder, with the risk increasing substantially in cases of comorbidity with major depressive disorder or other psychiatric conditions (9).

Additionally, anxiety sensitivity- defined as fear of anxiety-related symptoms and the cognitive concerns about the implications of these symptoms (e.g., fear of losing control or going “crazy”) has been linked to suicidal ideation and attempts (10, 11). In patients with panic disorder, the physical subfactor of anxiety sensitivity has also been associated with increased suicide risk (12). A meta-analysis by Stanley et al. (13) demonstrated a small to moderate association between anxiety sensitivity and suicidality, with the cognitive component being the most influential. In an extensive epidemiological study, Katz et al. (14) found a strong correlation between catastrophic interpretations of panic attacks and suicidal behavior in patients with major depressive disorder. Catastrophic cognitions measured by the cognitive subfactor of the Anxiety Sensitivity Index were shown to heighten the risk of suicidal ideation and attempts by increasing distress in response to these thoughts. Panic attacks and related symptoms may therefore represent independent suicide risk factors in patients with depressive disorders. However, Placidi et al. (15) reported that the co-occurrence of panic disorder and major depression does not appear to increase the lifetime risk of suicide attempts. They suggested that heightened anxiety, somatic preoccupations, and pronounced fear of death may function as protective factors against suicidal behavior. These findings are consistent with the Interpersonal-Psychological Theory of Suicidal Behavior which posits that individuals with high anxiety sensitivity are less likely to engage in life-threatening behavior due to their heightened fear of pain and death. In line with this, according to Hypothesis 3 of the Interpersonal Theory of Suicide, *‘the simultaneous presence of suicidal desire and a lowered fear of death serves as the condition under which suicidal desire transforms into suicidal intent’* (16).” Other studies suggest that anxiety disorders, especially severe panic, are independent risk factors for suicidal ideation beyond any co-occurring depressive symptoms, and that their co-occurrence with depression results in a compounded risk effect (17). Moreover, the comorbidity of anxiety and depression is recognized as a more significant risk factor for suicidality than depression alone, as anxiety disorders exacerbate the risk of suicide attempts (8). Panic-related fear of death has been shown to increase the odds of suicide attempts by sevenfold in depressed patients (18), and panic/agoraphobia comorbidity in major depression has been associated with an increased risk of suicidality, even when depression is controlled (8).

Hopelessness, defined as a negative outlook on oneself and the future, is another well-established risk factor for suicide. Some studies have shown that the relationship between depression and suicide diminishes or disappears when hopelessness is controlled for. Additionally, among previous suicide attempters, hopelessness is a stronger long-term predictor of repeat attempts than depression itself (19).

The present study aimed to assess the relative predictive power of depression, hopelessness, and anxiety-related factors (including panic, agoraphobia, overall anxiety, and anxiety sensitivity) in predicting suicide attempts in patients diagnosed with major depressive disorder (DD) and panic disorder with and without agoraphobia (PD/A). We hypothesized that in patients with DD and PD/A, depression severity would be a stronger predictor of suicide attempts than hopelessness or anxiety-related factors.

## 2 Methods

### 2.1 Study sample and recruitment

The study included 94 outpatients (N = 94), with 48 diagnosed with depressive disorder (DD) and 46 diagnosed with panic disorder with and without agoraphobia (PD/A). Treating psychiatrists diagnosed all patients based on the ICD-10 criteria (20) for current panic disorder with or without agoraphobia, or depressive disorder (single episode or recurrent, without psychotic features).

Exclusion criteria included: comorbidity of other major psychiatric disorders (psychotic disorders, bipolar disorder, substance use disorder, cognitive impairment or neurological conditions affecting assessment), severe medical illness that could interfere with psychological evaluation, inability to provide informed consent. Participants were recruited from the Clinic of Psychiatry, Clinical Centre of Serbia (N = 54), Community Health Care Kanarevo Brdo (N = 30), and Community Health Care Subotica (N = 10). The study was approved by the Institutional Review Boards of the participating medical institutions and by the Ethics Committee of the University Clinical Centre of Serbia.

### 2.2 Procedure

Patients were invited after ethical approval. All participants signed an informed consent form after receiving detailed information about the study, indicating their voluntary agreement to participate in the study. The selected self-report measures were administered by attending psychiatrists and psychologists. After providing consent, participants were given the questionnaires. Validated Serbian translations of the employed questionnaires were utilized in this research. Specifically, the Beck Depression Inventory II (BDI II) (21), the Anxiety Sensitivity Index 3 (ASI 3) (22), the Beck Anxiety Inventory (BAI) (23), the Beck Hopelessness Scale (BHS) (24), and the Beck Scale for Suicide Ideation (BSSI) (25) have all been psychometrically validated in Serbian samples, demonstrating satisfactory psychometric properties such as internal consistency, factor structure, and construct validity. The average time required to complete the instruments was approximately 30 minutes.

Participants completed seven self rated questionnaires, responding to a total of 139 items: 21 from the Beck Depression Inventory-II (BDI-II), 13 from the Panic and Agoraphobia Scale (PAS), 21 from the Beck Scale for Suicide Ideation (BSSS), 36 items from the Anxiety Sensitivity Index-Revised (ASI-R), 20 from the Beck Hopelessness Scale (BHS), and 21 from the Beck Anxiety Inventory (BAI). A total of 132 items were rated on a Likert-type scale reflecting participants’ level of agreement. An additional seven items, focusing on sociodemographic and clinical variables, required either the selection of predefined answers or written responses, depending on the item.

### 2.3 Assessment measures

Sociodemographic and Clinical Characteristics: A semi-structured questionnaire was developed specifically for the study to gather information about sex, age, education level, psychiatric diagnoses, and the number of past suicide attempts.

Beck Depression Inventory-II (BDI-II) (26): A 21-item self-report scale measuring depressive symptoms over the past week. It uses a 4-point Likert scale (0–3), with total scores ranging from 0 to 63, indicating the severity of depressive symptoms. It has adequate psychometric properties (Cronbach’s α = 0.88). The Beck Depression Inventory was chosen for this study due to its self-report nature, which allows individuals to report their subjective experience while focusing on the cognitive and emotional aspects of depression, particularly hopelessness and suicidal ideation, which were central to the goals of the study.

Panic and Agoraphobia Scale (PAS) (27): The scale assesses the severity of panic disorder symptoms, with or without agoraphobia. It includes 14 items rated on a 5-point scale (0–4), covering domains such as panic attacks, agoraphobia, anticipatory anxiety, functional disability, and health concerns. The total score ranges from 0 to 52. The scale has good internal consistency (α = 0.78) and test-retest reliability (r = 0.73). The participants rated the severity of their panic/agoraphobic symptoms over the previous week.

Beck Scale for Suicide Ideation (BSSI) (28): This instrument assesses the intensity of suicidal thoughts, attitudes, and plans over the previous seven days. It comprises 21 items, of which 15 are rated on a 3-point scale (0–2) and 6 on a 4-point scale (0–3). Total scores range from 0 to 48. The scale demonstrates excellent internal reliability (Cronbach’s α = 0.94) and shows strong correlations with the Beck Depression Inventory (r = 0.75).

Beck Hopelessness Scale (BHS) (29): A 20-item instrument measuring negative expectations about the future and a perceived inability to change negative outcomes. Items are answered as true or false, with total scores ranging from 0 to 20. Higher scores indicate greater hopelessness. The scale has strong psychometric properties, including a reliability coefficient of 0.91 in psychiatric populations. It assesses negative expectations about oneself and the future, based on participants’ current cognitive appraisal.

Anxiety Sensitivity Index – Revised (ASI-R) (30): A 36-item self-report measure assessing fear of anxiety-related sensations due to beliefs about their potentially harmful consequences. The total score ranges from 0 to 144. Subscales assess fear related to respiratory symptoms, public anxiety reactions, cognitive control, and cardiovascular symptoms. The ASI-R measures a stable, trait-like tendency to fear anxiety-related sensations and the potential consequences of anxiety, rather than assessing symptom severity within a short time frame. The scale demonstrates high internal consistency (α = 0.94).

Beck Anxiety Inventory (BAI) (31): A 21-item scale measuring the severity of self-reported anxiety symptoms. Items are rated on a 4-point scale from 0 (not at all) to 3 (severely). Participants rated how much each symptom had bothered them over the past week. Total scores range from 0 to 63, with higher scores indicating more severe anxiety. The instrument has strong internal consistency (α = 0.92) and good test-retest reliability (r = 0.75).

### 2.4 Statistics

Data were analyzed using IBM SPSS Statistics 22. Descriptive statistics were used to summarize the socio-demographic characteristics of the participants and the scores on the various scales. Internal consistency of the measures was assessed using Cronbach’s α. Correlation analyses were conducted to determine relationships between the scores of the different instruments. Canonical discriminant analysis was performed to develop a predictive model for suicide attempts. In this analysis, cases with missing data on any predictor variable were excluded listwise, which resulted in two PD/A participants being removed from the validation sample (final N = 44).

## 3 Results

### 3.1 Descriptive statistics of the study sample

In the DD group, the patients were predominantly female (34 versus 14), with a mean age of 49.37 years (SD = 11.45). Educational attainment was as follows: high school 47.9% (N = 23), elementary school 16.0% (N = 8), university 14.9% (N = 7), and college 8.5% (N = 4). Educational data were unavailable or unclassified for 12.5% of participants (*N* = 6). The mean duration of the disorder was 9.58 years (SD = 7.08).

The majority of the PD/A patient sample were female (35 versus 11), with a mean age of 39.83 years (SD = 11.84). Educational attainment in this group was 73.9% high school (N = 34), 17.14% university (N = 8), 6.5% elementary school (N = 3), and 2.2% college (N = 1). The mean duration of the disorder was 5.35 years (SD = 5.18).

The two patient groups differed significantly in terms of age (p < 0.05), illness duration (p < 0.05), and education level (Chi-square test, three df = 12.27, p < 0.001).

### 3.2 Descriptive statistics of the applied instruments

Descriptive statistics for the applied instruments in both patient groups are presented in **Table 1**.

**Table 1:** Descriptive statistics of the administered instruments (means, standard deviations, and group differences).

	Diagnosis			
	Panic disorder		Depressive disorder	
	Mean	SD	Mean	SD
Anxiety Sensitivity Index – Revised (ASI-R);	78.61_a_	33.132	71.29_a_	36.572
Beck Anxiety Inventory (BAI)	34.13_a_	16.180	32.40_a_	13.470
Beck Depression Inventory-II (BDI-II)	23.96_a_	15.210	36.90_b_	13.170
Beck Hopelessness Scale- (BHS)	9.09_a_	7.162	13.60_b_	6.059
Beck Scale for Suicide Ideation (BSSI)	2.26_a_	3.986	10.50_b_	11.495
Panic and Agoraphobia Scale (PAS)	23.73_a_	9.066	15.90_b_	9.377

Groups are statistically significantly different at *p* < 0.05 if they do not share the same subscript (a or b) on the same variable. Bonferroni correction for multiple comparisons was applied.

As expected, patients diagnosed with DD had significantly higher scores on the Beck Depression Inventory (p < 0.05), the Beck Scale for Suicide Ideation (p < 0.05), and the Beck Hopelessness Scale (p < 0.05). In contrast, patients with PD/A scored significantly higher on the Panic and Agoraphobia Scale (p < 0.05). No significant differences were observed between the two groups on the Beck Anxiety Inventory or the Anxiety Sensitivity Index–Revised.

### 3.3 Correlation analysis of the applied instruments

**Table 2** shows the correlations between the scores of the applied instruments across both patient samples:

**Table 2:** Correlation analysis of the administered instruments in patients with PD/A (above the diagonal) and DD (below the diagonal).

	BSSI	ASI-R	BAI	BDI-II	PAS	BHS
BSSI	1	.616**	.526**	.727**	.360*	.689**
ASI-R	.049	1	.680**	.768**	.671**	.653**
BAI	.294*	.652**	1	.634**	.716**	.408**
BDI-II	.763**	.197	.449**	1	.712**	.836**
PAS	.352*	.246	.401**	.424**	1	.518**
BHS	.771**	.276	.437**	.744**	. 544**	1

*Correlation is significant at the 0.01 level (1-tailed); ** Correlation is significant at the 0.05 level (2-tailed); BSSI—Beck Scale for Suicide Ideation; ASI-R—Anxiety Sensitivity Index-Revised; BAI—Beck Anxiety Inventory; BDI-II—Beck Depression Inventory-II; PAS—Panic and Agoraphobia Scale; BHS—Beck Hopelessness Scale.

In the DD group, ASI-R scores were significantly correlated only with the Beck Anxiety Inventory (BAI), whereas their associations with the BDI-II, PAS, BHS, and BSSI were small and did not reach statistical significance. In contrast, in the PD/A group, ASI-R scores showed a broader pattern of significant correlations with the other clinical measures.

### 3.4 Prediction equation for suicide attempts

In the next step of data analysis, Canonical Discriminant Analysis was applied to the sample of patients with DD to develop a predictive equation for suicide attempts. The canonical correlation was 0.765 (p < 0.001). The means, standard deviations (SD), standardized canonical discriminant function coefficients (W), and structure coefficients (f) for patients with DD are presented in **Table 3**.

**Table 3:** Means, standard deviations (SD), standardized canonical discriminant function coefficients (W), and structure coefficients (f) for patients with DD.

Assessment Measures	Suicide attempt					
	Without attempt		With attempt			
	Mean	SD	Mean	SD	W	f
Beck Scale for Suicide Ideation	2.80_a_	4.397	18.87_b_	10.981	.691	.838
Beck Depression Inventory-II	28.48_a_	9.138	46.04_b_	10.559	.514	.766
Beck Hopelessness Scale	10.52_a_	5.501	16.96_b_	4.781	.009	.535
Beck Anxiety Inventory	27.84_a_	11.618	37.35_b_	13.826	.221	.321
Anxiety Sensitivity Index-Revised	66.00_a_	33.132	77.04_a_	39.918	.003	.130
Panic and Agoraphobia Scale	14.80_a_	9.866	17.09_a_	8.878	-.473	.104
Canonical function centroids	-1.116		1.213			

According to the structure coefficients (f), scores on the Beck Scale for Suicide Ideation were the strongest predictors of suicide attempts, followed by scores on the Beck Depression Inventory and the Beck Hopelessness Scale. The standardized canonical discriminant function coefficients (W) indicated that the Beck Hopelessness Scale and the Anxiety Sensitivity Index–Revised were redundant and could be excluded from a suicide risk assessment battery.

Interestingly, scores on the Panic and Agoraphobia Scale demonstrated a suppressor effect. When controlling for all other variables included in the analysis, panic and agoraphobic symptoms reduced the likelihood of a suicide attempt by 11%.

Receiver Operating Characteristic (ROC) curve analysis revealed that the optimal cut-off score for the Beck Scale for Suicide Ideation is 11, while the optimal cut-off score for the Beck Depression Inventory is 35.

### 3.5 Sensitivity of the battery test for suicide attempt prediction

The canonical discriminant function was derived in the DD group and achieved a correct classification rate of 87.5%, with a sensitivity of 82.6% for patients with a history of suicide attempt and a specificity of 92.0% for those without (“Cases Selected”) (**Table 4**).

**Table 4:** Sensitivity of the test battery in predicting suicide attempts.

			Suicide attempt	Predicted Group Membership		Total
				no	yes	
Cases Selected	Original	Count	no	23	2	25
			yes	4	19	23
		%	no	**92.0**	**8.0**	100.0
			yes	**17.4**	**82.6**	100.0
Cases Not Selected	Original	Count	no	39	4	43
			yes	1	0	1
		%	no	**90.7**	**9.3**	100.0
			yes	**100.0**	**.0**	100.0

**a.** The discriminant function correctly classified 87.5% of the selected original grouped cases. **b.** The discriminant function correctly classified 88.6% of the unselected original grouped cases.

‘Cases Selected’ refers to the DD derivation sample, whereas ‘Cases Not Selected’ refers to the PD/A validation sample. Two PD/A patients were excluded listwise due to missing data on predictor variables (final N = 44).”

Percentages (%) are shown in bold.

The same function was subsequently applied without reestimating coefficients to the PD/A group as an independent validation sample (“Cases Not Selected”). After listwise exclusion of two PD/A patients with missing predictor data, the final validation sample included 44 cases.

The model yielded an overall accuracy of 88.6%. However, the model failed to correctly identify the only PD/A patient with a prior suicide attempt (sensitivity = 0%; specificity = 90.7%). In the PD/A validation sample, two patients were excluded listwise due to missing predictor data, resulting in 44 cases.

Notably, the model predicted that 9.3% of PD/A patients were at elevated risk for a suicide attempt, despite the fact that none of them (except the single case missed by the model) had an actual suicide attempt history.

## 4 Discussion

The results of this study confirm that high scores on the Beck Scale for Suicidal Ideation (BSSI) and the Beck Depression Inventory-II (BDI-II) are the strongest predictors of suicide attempts in patients with Depressive Disorder (DD). These findings are consistent with previous research linking increased suicidality with higher levels of depressive symptom severity (26, 32, 33). Our analysis of the Receiver Operating Characteristic (ROC) curve revealed optimal cut-off scores for predicting suicide attempts: 11 for the BSSI and 35 for the BDI-II. These thresholds may provide useful benchmarks for clinical screening and risk stratification.

Interestingly, panic/agoraphobia symptoms had a suppressor effect on suicide attempts in patients with DD. The cognitive and behavioral components of anxiety associated with panic and agoraphobia appeared to serve as a protective factor, possibly due to increased fear of death, cognitive patterns marked by catastrophic misinterpretation of somatic cues, heightened interoceptive awareness, or greater treatment-seeking behavior. Safety behaviors, such as avoiding certain places, carrying medication, or going outside only when accompanied by a trusted person, reflect a strong drive for self-preservation and avoidance of perceived danger. These patients often exhibit a heightened desire to survive rather than to die, despite experiencing extreme distress. Goodwin & Hoven (34) found that individuals with panic disorder are more likely to be in contact with the healthcare system during times of crisis. This supports the idea that specific anxiety symptoms, though distressing, may inhibit suicidal action in individuals with high anxiety sensitivity or fear of harm. These findings align with the work of Placidi et al. (15), who demonstrated that comorbid panic disorder in MDD patients does not increase the lifetime risk of suicide attempts. However, this contrasts with studies suggesting that comorbid anxiety disorders amplify the risk of suicidal ideation and attempts beyond depression alone (8, 13, 17). One possible explanation for these discrepancies lies in the heterogeneity of anxiety presentations and in the differential roles played by specific anxiety dimensions (e.g., physiological *vs* cognitive).

Further analysis showed that scores on the Beck Hopelessness Scale (BHS) and Anxiety Sensitivity Index-Revised (ASI-R) were redundant and did not significantly contribute to the prediction of suicide attempts. This suggests that, in this sample, screening for depression severity and suicidal ideation may be sufficient for identifying high-risk individuals, potentially streamlining assessment protocols in high-demand clinical settings. This finding contrasts with earlier studies (e.g., 19) that identified hopelessness as a key predictor of suicide attempts, as well as research emphasizing the importance of anxiety sensitivity (13, 14). Differences in sample characteristics, measurement tools, or statistical modelling may account for the divergent findings.

The discriminant function demonstrated strong predictive performance in the DD group, with high sensitivity (82.8%) and specificity (92%), but that when applied to the PD/A group, the apparently high overall accuracy is largely driven by the correct classification of non-attempters. We emphasize that in the PD/A group, the function does not correctly classify the single attempter (sensitivity = 0%). We interpret the four “high-risk but non-attempting” PD/A cases in light of our previous study in a partly overlapping sample (35), which showed that patients with panic disorder/agoraphobia exhibited lower suicidality than patients with MDD, and that panic severity did not independently increase suicide risk once depression was controlled. This supports the interpretation that prominent panic/agoraphobic symptoms may, in some patients, inhibit the translation of depressive distress into suicidal behavior. At the same time, we clearly state that, from usefulness for individual risk stratification, and that the discriminant solution for PD/A should therefore be considered preliminary and hypothesis-generating rather than a clinically ready tool.

From a theoretical perspective, our findings resonate with the Interpersonal-Psychological Theory of Suicide, which posits that a heightened fear of death or pain may lower the acquired capability for suicide. In line with this, according to Hypothesis 3 of the Interpersonal Theory of Suicide, *‘the simultaneous presence of suicidal desire and a lowered fear of death serves as the condition under which suicidal desire transforms into suicidal intent’* (16).” Individuals with panic disorder may experience increased suicidal ideation due to psychological pain or perceived burdensomeness, but may be less likely to act due to anxiety-related inhibitions. This may explain the observed suppressor effect of panic/agoraphobia symptoms.

Clinically, the study suggests that in patients with DD, assessing suicidal ideation and depressive severity is of primary importance. Anxiety-related measures such as hopelessness or anxiety sensitivity may be less critical for suicide risk stratification in this population. In contrast, for patients with PD/A, the model derived from the MDD group may help flag those at elevated risk, particularly in the presence of depressive symptoms.

## 5 Conclusion

Elevated levels of depressive symptomatology and suicidal ideation emerged as the most robust predictors of suicide attempts among patients with DD, whereas panic and agoraphobic symptoms exerted a suppressor effect. These findings highlight the importance of rigorous clinical assessment of both depression severity and suicidal ideation in the management and prevention of suicidal behavior in patients diagnosed with DD. In the PD/A group, the model generated elevated estimated risk scores but failed to identify the single suicide attempter, yielding zero sensitivity. Consequently, the predictive utility in PD/A remains limited, and these results should be considered preliminary. Replication in larger PD/A samples with higher base rates of suicide attempts is needed to evaluate the generalizability and clinical applicability of these findings.

## 6 Limitations of the study

Several limitations of this study should be acknowledged. First, it relied solely on self-report measures, which may be subject to response bias and limited insight. Second, the cross-sectional design prevents conclusions about causality or changes in suicide risk over time. Third, although adequate for multivariate analyses, the sample size may restrict generalizability to broader populations and specific diagnostic subgroups. Future research should replicate these findings using longitudinal designs and larger, more diverse samples, ideally incorporating behavioral and biological measures of suicidality.

An additional limitation is the very low number of patients with panic disorder/agoraphobia (PD/A) who had a history of suicide attempt. Consequently, the classification performance in the PD/A group was driven almost entirely by the correct classification of non-attempters, resulting in 0% sensitivity for identifying the single PD/A attempter. This severely limits the clinical utility of the model for this subgroup, and the findings should therefore be considered preliminary and interpreted with caution until replicated in larger samples with a higher base rate of suicidal behavior.

The study also did not control for all psychiatric comorbidities. Previous research indicates that disorders such as borderline personality disorder, obsessive–compulsive disorder, and other mood and anxiety disorders may substantially increase suicide risk in both panic disorder and depressive disorder. Further studies should also incorporate other psychological constructs, such as impulsivity, aggression, and psychological pain, which have been identified as relevant mediators of suicidality.

Finally, the study did not distinguish between panic disorder with and without agoraphobia, although these diagnostic categories are separated in DSM-5 (36). Future research in our country should differentiate between uncomplicated panic disorder, panic disorder with comorbid agoraphobia, and isolated agoraphobia to more accurately evaluate the relationship between these conditions and suicidality.

## Data Availability

The raw data supporting the conclusions of this article will be made available by the authors, without undue reservation.
